# Facts Establishing the Deleterious Properties of Rice, &c.

**Published:** 1834-01-01

**Authors:** 


*Facts establishing the Deleterious Properties of Rice used as an Article of Food.* By Robert Tytler, m.d.—London, 1833. 8vo. pp. 60.

"Bad rice is a very bad thing," says Dr. Tytler; and we agree with him: "but," cries the great Anti-Oryzeus, "bad rice is the cause of the cholera;" and here, we, in common with all the sane and sober, dissent. The importations of spoiled rice, cankered enough to offer on the altar of Rubigo herself, began long before 1831, when the Asiatic cholera first appeared in these kingdoms. But enough of this. We love novelty, and are therefore delighted with the honours which Dr. Tytler has accumulated on his title-page. The letters which modern authors usually append to their names are expensive luxuries; for, to buy a life-interest in these symbols costs, on an average, 10*l.* 9*s.* 9¼*d.* per letter, as we are informed by Mr. Babbage. Now the Tytlerian titles cost nothing, but, on the contrary, are the evidence of many a rupee which has descended into our author's pockets. Here they are: "Surgeon in the Honourable East India Company's service; formerly assistant-surgeon of his Majesty's 81st regiment of foot, chief surgeon of Fort Marlborough and its dependencies; acting superintending surgeon of the south-eastern division of the Bengal army, during the campaign in Arracan, in 1825; acting superintending surgeon of the Dinapore division of the Bengal army; and senior surgeon in the field during the recent campaigns of 1832, 1833, against the Coles and Choars, in Chota Nagpore, and the Jungle Mehals." And we have ventured to differ from a man who has faced the Coles and Choars in Chota Nagpore, and the Jungle Mehals! We tremble at our temerity.

